# Constrained acetabular liner total hip arthroplasty versus conventional hemiarthroplasty for unstable comminuted femoral intertrochanteric fractures in elderly patients with neurological disorders

**DOI:** 10.1186/s12893-026-03973-2

**Published:** 2026-06-23

**Authors:** Mingming Dong, Yandan Liu, Congcong Wei

**Affiliations:** 1 Department of Geriatrics, No. 215 Hospital of Shaanxi Nuclear Industry, Xianyang, Shaanxi P.R. China; 2 Department of Xi’an, Peihua University, Xi’an, Shaanxi P.R. China; 3 Department of Joint Surgery, No. 215 Hospital of Shaanxi Nuclear Industry, No.35 Weiyang West Road, Xianyang, Shaanxi P.R. China

**Keywords:** Femoral intertrochanteric fractures, Constrained acetabular liner, Total Hip Arthroplasty, Hip arthroplasty, Hemiarthroplasty, Neurological disorders

## Abstract

**Background:**

To evaluate whether constrained acetabular liners (CAL) used in total hip arthroplasty (THA) reduce the risk of dislocation and related complications in patients with neuromuscular disorders and unstable comminuted femoral intertrochanteric fractures (ITF).

**Methods:**

This retrospective cohort study included patients diagnosed with Evans type III-V ITFs who underwent hemiarthroplasty or THA at our institution from July 2011 to July 2016. Patients were divided into two groups: a hemiarthroplasty group and a CAL group. Radiological data, clinical records, and laboratory test results were meticulously collected. Differences between the two groups were analyzed to support clinical decision-making regarding treatment strategies.

**Results:**

Significant differences were observed between the two groups in operation duration (90.50 ± 25.12 vs. 110.10 ± 26.38 min, *P* = 0.008), intraoperative blood loss (205.12 ± 68.50 vs. 275.58 ± 83.36 mL, *P* = 0.003) and blood transfusion rate (16.67% vs. 30.87%, *P* = 0.012). Compared to hemiarthroplasty, CAL was associated with a significantly higher risk of heterotopic ossification (HO) (Odds ratio = 1.198, 95% CI: 1.020–1.850), a higher risk of aseptic loosening (Odds ratio = 1.155, 95% CI: 0.708–1.782), and a lower risk of dislocation (Odds ratio = 0.120, 95% CI: 0.022–0.552).

**Conclusions:**

Among patients with neurological disorders undergoing HA for unilateral unstable comminuted femoral ITF, CAL THA was correlated with a lower postoperative dislocation rate but increased risks of aseptic loosening and HO. Despite reducing dislocation risk, the overall clinical benefits of CAL appeared limited. Additionally, no significant differences in long-term hip function outcomes were observed between CAL and conventional hemiarthroplasty.

**Supplementary Information:**

The online version contains supplementary material available at https://doi.org/10.1186/s12893-026-03973-2.

## Introduction

Femoral intertrochanteric fractures (ITFs), often caused by simple household falls, constitute the most common fracture type among elderly individuals with osteoporosis [1]. Patients suffering from ITFs typically experience increased rates of complications and mortality. This increase primarily results from advanced age, poor bone quality, and multiple comorbid conditions [2]. Previous studies reported approximately 30% mortality within the first 12 months following injury [3]. Internal fixation (IF) is usually considered the preferred method, as it enables rapid recovery of previous functional levels and has low complication and mortality rates [4]. However, IF may not always achieve therapeutic goals, particularly for ITFs characterized by unstable fracture patterns (Evans types III, IV, and V), extensive comminution, and compromised bone quality. Under these conditions, achieving adequate fracture reduction and stable fixation can be challenging [5].

Hip arthroplasty (HA) has emerged as a favored alternative to IF due to potential benefits, such as enabling patients to bear full weight immediately after surgery [6]. Prosthetic dislocation is a common complication following THA, with soft tissue tension significantly influencing its occurrence [7]. Ogawa [8] reported approximately a 25% difference in soft tissue tension between patients experiencing recurrent dislocations after THA and those without dislocations. In patients with neurological disorders, the risk of dislocation may increase due to muscular laxity or muscle imbalance around the hip [9]. Reported dislocation rates following primary THA range from 0.2% to 10% within the first postoperative year [10]. However, patients with neuromuscular conditions have exhibited dislocation rates as high as 13% [11]. The use of constrained acetabular liners (CAL) may significantly reduce the risk of recurrent dislocation by increasing stability [12]. However, it remains unclear whether CAL implantation raises the risk of postoperative complications, potentially affecting limb function. Therefore, this study aims to investigate whether CAL reduces the risk of dislocation and associated complications in patients with neuromuscular disorders. Radiological, clinical, and laboratory data were comprehensively collected from patients with neurological disorders who underwent total HA or hemiarthroplasty for unstable comminuted femoral ITF. Differences between these patient groups were analyzed to inform clinical decision-making regarding treatment strategies.

## Patients and methods

### Study design

This retrospective study included patients diagnosed with Evans type III-V femoral ITF who underwent hemiarthroplasty or THA at No. 215 Hospital of Shaanxi Nuclear Industry between July 2011 and July 2016. The final follow-up was completed in May 2022. Patients were divided into two cohorts: those who underwent total HA with CAL and those who received hemiarthroplasty.

### Participants

The inclusion criteria were as follows: (1) patients with complex and unstable ITF (Evans types III, IV, and V); (2) patients with quadriceps muscle weakness caused by neuromuscular disorders, such as Parkinson’s syndrome, cerebral palsy, and cerebrovascular disease, before hip replacement surgery; (3) patients with a follow-up duration of more than 24 months; and (4) patients aged 50–79 years. Patients aged 50–59 years with neurological disorders were also included because they often present with osteoporosis, reduced physical function, and an increased risk of fracture-related complications. Therefore, this high-risk middle-aged population was analyzed together with patients aged 60–79 years. This age criterion was based on single-center clinical experience and does not represent a universal orthopedic definition of elderly patients.

The exclusion criteria were as follows: (1) follow-up duration of less than 24 months; (2) femoral ITF associated with primary or metastatic malignant tumors or other pathological fractures; (3) previous hip surgery performed for other reasons; and (4) incomplete medical records or plain radiographs. Incomplete medical records referred to missing key information regarding preoperative comorbidities, neurological function grading, intraoperative findings, or postoperative follow-up data. Incomplete plain radiographs referred to unavailable anteroposterior pelvic and bilateral lower extremity radiographs, unclear fracture classification images, or absence of complete preoperative, postoperative, and follow-up radiographic data required for outcome evaluation.

This study was reviewed and approved by the Institutional Review Board of No. 215 Hospital of Shaanxi Nuclear Industry (Approval No. 2026-003) and was conducted in accordance with the Declaration of Helsinki and the Health Insurance Portability and Accountability Act regulations. In this retrospective study, all patient data were fully de-identified and anonymized before analysis. No personally identifiable information was retained, and no direct participant contact or intervention was performed. Therefore, the requirement for written informed consent was formally waived by the Institutional Review Board.

## Data collection

### Demographic information

Patient demographics and general information were collected from medical records. These included age, gender, body mass index (BMI), muscle strength of the affected lower limb, side of surgery, hospitalization duration, bone mineral density, and follow-up period.

### Clinical evaluations

**Fig. 1:**
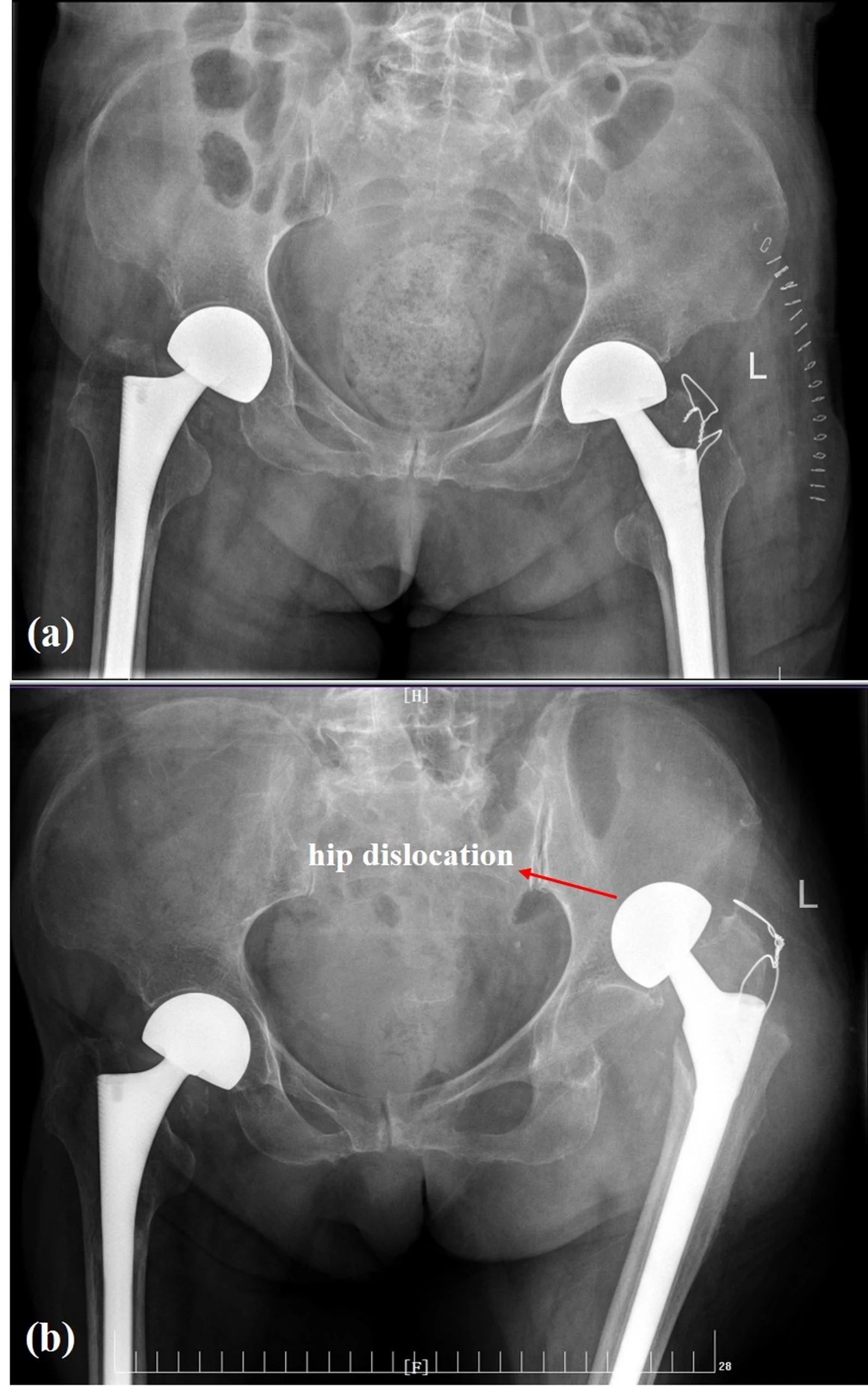
Radiographs of a female patient aged 60–70 years with left femoral intertrochanteric fracture treated with hemiarthroplasty. **a** Postoperative radiograph at discharge: no obvious periprosthetic abnormality. **b** Radiograph obtained 2 years postoperatively: hip dislocation

**Fig. 2:**
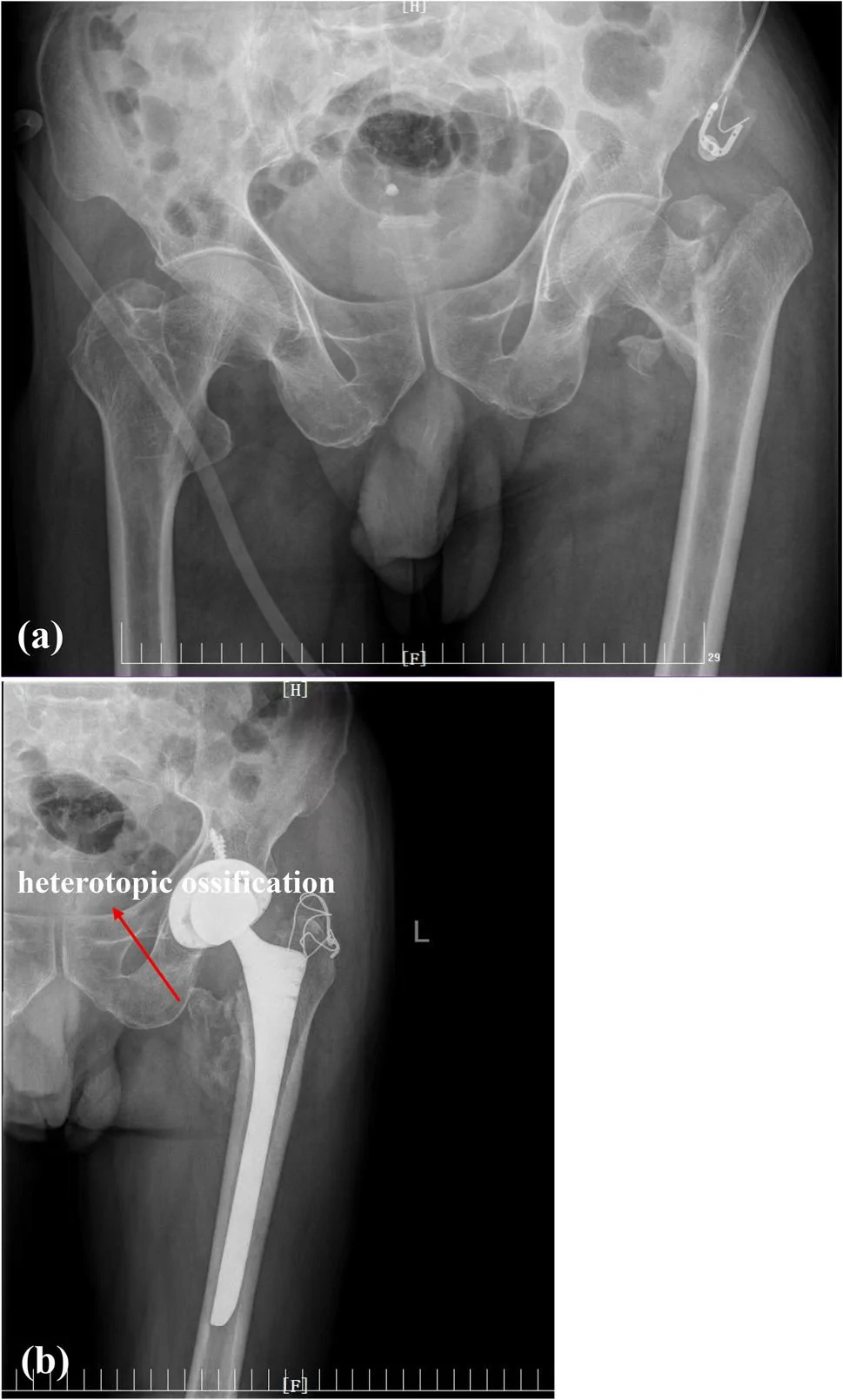
Images of a male patient aged 70–80 years with left hip femoral intertrochanteric fracture. **a** Postoperative radiograph. **b** Radiograph 3 years after surgery: severe periprosthetic heterotopic ossification

To evaluate prognosis and differences between the hemiarthroplasty and constrained acetabular liner groups, the Harris Hip Score (HHS), Visual Analogue Scale (VAS), WOMAC scores, and Forgotten Joint Score (FJS) were recorded at 12 and 24 months postoperatively. Perioperative indicators and complications evaluated included operation duration, intraoperative blood loss, blood transfusion, incision length, postoperative delirium, poor wound healing, periprosthetic femoral fracture, dislocation (Fig. 1), aseptic loosening, infection, deep vein thrombosis, death within one year, and HO (Fig. 2). HO was assessed using the Brooker grading system. Radiographic evaluations were conducted in a single-blind manner by two senior orthopedic surgeons who were blinded to group allocation. Blood transfusion was initiated when hemoglobin concentration fell below 8 g/dL, or below 10 g/dL in the presence of clinical signs of hypovolemia.

Aseptic loosening was defined by radiographic signs of osteolysis around the prosthesis, accompanied by clinical manifestations such as hip pain and limited range of motion. Symptoms indicative of aseptic loosening include pain during weight-bearing activities or joint movement, often located in the thigh or groin region. Radiographic assessment typically shows osteolysis, radiolucent lines, cup migration, or stem subsidence exceeding 2 mm [13].

### Radiographic evaluation

All patients underwent anteroposterior X-ray examinations of the pelvis and both lower extremities before and after surgery. Computed tomography (CT) scans of both hips were performed postoperatively. An experienced orthopedic surgeon obtained measurements using standardized anatomical landmarks through the hospital’s image archiving system. Radiological indicators measured included leg length discrepancy (LLD), femoral offset, femoral stem anteversion, and stem alignment: (1) LLD: LLD was measured as the perpendicular distance from a horizontal line connecting the teardrop points to the most prominent part of the lesser trochanter. (2) Femoral Offset: Measured as the horizontal distance between the center of hip rotation and the femoral shaft axis. (3) Femoral stem anteversion: Measured as the angle between the coronal axis and the extended centerline of the femoral stem neck on transverse CT images. (4) Femoral stem coronal alignment: Assessed using X-ray images, measuring the angular deviation between the stem axis and proximal femoral axis on the coronal plane. Stem alignment was classified as varus, neutral, or valgus based on deviation direction.

### Surgical procedure

Surgical intervention was performed after disease stabilization. The same surgical and nursing teams conducted all operations. Patients were positioned in the lateral decubitus position, and surgery was performed using a posterolateral approach. A surgical incision exposed the hip joint. The skin and subcutaneous tissues were dissected to expose external rotator muscles. The joint capsule was then opened. The femoral head and neck were carefully removed, preserving the greater trochanter, rotator muscles, and bone fragments. Osteotomy was performed 1–1.5 cm superior to the lesser trochanter, extending to the junction of the femoral neck and greater trochanter, after which the femoral head was excised.

### CAL-THA group

Patients in the CAL group received cementless hydroxyapatite-coated stems, cementless cups, ceramic ball heads, and highly cross-linked polyethylene (HXLPE) liners (AK MEDICAL, China). In the CAL group, the femoral medullary canal was reamed to the appropriate size. Subsequently, meticulous acetabular reaming was performed, removing acetabular cartilage and subcortical bone, while preserving subchondral and cancellous bone structures. A suitable acetabular component and CAL were implanted into the prepared cavity. Trial reductions were performed to determine optimal tissue tension, abductor muscle balance, and equal leg lengths. During trials, neck length, offset, and version were carefully restored to ensure hip joint stability. After trial reduction, the femoral stem prosthesis and ceramic head were implanted, and the hip joint was reduced. Following hip reduction, greater trochanter fragments were stabilized using tension-band wiring techniques, depending on fragment size. 18-gauge stainless steel wires folded into double rows were passed through the lesser trochanter, medial proximal femur, and iliopsoas muscle with a wire passer. The gluteus medius muscle attached to the greater trochanteric fragment was secured using a figure-eight wiring configuration and tension-band wiring technique [14]. Wires were tightened gradually to generate interfragmentary compression, avoiding excessive tension to prevent bone cut-out or wire loosening.

### Hemiarthroplasty group

Patients in the hemiarthroplasty group received a cementless femoral stem and bipolar head (AK MEDICAL, China). Following metaphyseal preparation of the femur, the anatomical prosthesis and bipolar head were implanted.

All patients received 1.5 g cefuroxime sodium intravenously 30 min before surgery for infection prophylaxis. Postoperatively, 1.5 g cefuroxime sodium was administered intravenously twice daily until 24 h after surgery. During hospitalization, patients received 5000 IU low-molecular-weight heparin sodium subcutaneously once daily. After discharge, patients continued anticoagulation with oral rivaroxaban (10 mg once daily) until postoperative day 35.

Postoperative rehabilitation after hip replacement generally comprises three phases. Phase I lasts approximately six weeks, emphasizing tissue protection and wound healing. Additional goals during Phase I include restoration of joint mobility, independent gait, daily activity performance, and neuromuscular function improvement. Phase II typically extends from weeks 6 to 12 postoperatively, focusing on further improving range of motion, strength, and mobility. The final rehabilitation phase occurs between 12 and 24 weeks after surgery, concentrating on enhancing aerobic fitness, muscular strength, and endurance [15].

### Statistical analysis

Statistical analyses were performed using Excel 2016 (Microsoft Corporation, Seattle, WA, USA) and SPSS 19.0 (IBM, Armonk, NY, USA). Normality tests were conducted for all continuous variables before comparisons. Continuous variables with normal distribution were presented as mean ± standard deviation, and comparisons were made using Student’s t-test. Non-normally distributed continuous variables were expressed as median (interquartile range) and analyzed using the Mann-Whitney U test. Categorical variables were expressed as frequencies and analyzed using the chi-square test. Statistical significance was defined as *P* < 0.05.

## Results

### Baseline characteristics of patients

A total of 306 hips underwent hemiarthroplasty, and 298 hips received CAL. No significant differences in demographic or baseline characteristics were observed between the two groups. Characteristics included sex, age, body mass index, muscle strength of the affected lower limb, Evans type, side of surgery, hospitalization duration, prefracture functional status, bone mineral density, and follow-up duration. Baseline patient characteristics are summarized in Table 1.

**Table 1:** Demographic features of the patients

Patient characteristics	Hemiarthroplasty group (n=306)	Constrained acetabular liners group (n=298)	t-value, χ² value	P
Sex				
Male	122(39.87%)	99(33.22%)	χ² = 0.253	0.561
Female	184 (60.13%)	199(66.78%)		
Age (years)	59.34±3.56	60.40±4.23	t = 0.598	0.956
Body Mass Index (kg/m2)	25.01±2.32	25.15±2.11	t = 0.934	0.323
Muscle strength of the affected lower limb				
Level 3	158(51.63%)	152(51.01%)	χ² = 0.216	0.785
Level 4	148(48.37%)	146(48.99%)		
Evans type				
Type III	51(16.67%)	47(15.77%)	χ² = 2.236	0.773
Type IV	82(26.80%)	65(21.81%)		
Type V	173(56.53%)	186(62.42%)		
Side of surgery				
Left	151(49.35%)	148(49.66%)	χ² = 0.102	0.153
Right	155(50.65%)	150(50.34%)		
Hospitalization time (days)	11.7±2.3	12.0±1.5	t = 0.106	0.373
Prefracture functional status				
Uses an assistive device for walking	80(26.14%)	75(25.17%)	χ² = 0.372	0.259
Able to ambulate without assistive device	306(73.86%)	223(74.83%)	χ² = 0.378	0.520
Bone mineral density	−3.0 ± 0.5	−3.4 ± 0.6	t = 0.375	0.601
Follow-up (months)	73.26±5.56	70.17±5.51	t = 1.713	0.101

### Clinical outcomes and complications

No significant differences were noted between groups regarding postoperative delirium, poor wound healing, infection, deep vein thrombosis, or death within one year. However, significant differences existed in operation duration (90.50 ± 25.12 vs. 110.10 ± 26.38 min, *P* = 0.008), intraoperative blood loss (205.12 ± 68.50 vs. 275.58 ± 83.36 mL, *P* = 0.003), and blood transfusion rate (16.67% vs. 30.87%, *P* = 0.012). Among the 604 patients, 140 (23.2%) developed HO. According to Brooker classification, 13.7% were grade I, 8.3% grade II, and 1.2% grade III. The incidence of HO was significantly higher in the CAL group (30.20% vs. 16.34%, *P* = 0.021). Elderly patients with femoral ITF and neurological disorders had a significantly higher dislocation rate in the hemiarthroplasty group compared to the CAL group (8.50% vs. 1.01%, *P* = 0.010). Aseptic loosening occurred significantly less frequently in the hemiarthroplasty group compared to the CAL group (3.27% vs. 10.07%, *P* = 0.032). Patient clinical characteristics are summarized in Table 2. After adjusting for age, operation duration, intraoperative blood loss, and blood transfusion, the CAL procedure was associated with a significantly higher risk of HO (Odds ratio = 1.198, 95% CI: 1.020–1.850), increased aseptic loosening (Odds ratio = 1.155, 95% CI: 0.708–1.782), and lower dislocation risk (Odds ratio = 0.120, 95% CI: 0.022–0.552) compared to hemiarthroplasty (Table 3).

**Table 2:** Perioperative indicators and complications in patients in hemiarthroplasty group and constrained acetabular liners group

	Hemiarthroplasty group (n=306)	Constrained acetabular liners group (n=298)	t-value, χ² value	P
Duration of operation (minutes)	90.50 ± 25.12	110.10 ± 26.38	t = -1.368	0.008
Intraoperative blood loss (mL)	205.12±68.50	275.58±83.36	t = -3.536	0.003
Blood transfusion	51(16.67%)	92(30.87%)	χ² = -1.230	0.012
Incision length (cm)	11.8±0.8	12.5±0.5	t = 1.373	0.505
Postoperative delirium	34(11.11%)	44(14.77%)	χ² = 0.820	0.536
Poor wound healing	9(2.94%)	6(2.01%)	χ² = 0.670	0.536
Periprosthetic femoral fracture	7(2.28%)	8(2.68%)	χ² = 0.986	0.863
Dislocation	26(8.50%)	3(1.01%)	χ² = 5.155	0.010
Aseptic loosening	10(3.27%)	30(10.07%)	χ² = 4.320	0.032
Infect	2(0.65%)	5(1.68%)	χ² = 0.253	0.583
Deep vein thrombosis	37(12.09%)	50(16.78%)	χ² = 0.703	0.150
Deaths within one year	43(14.05%)	39(13.09%)	χ² = 1.011	0.480
Heterotopic ossification				
I	28	55		
II	20	30		
III	2	5		
IV	0	0		
Total	50(16.34%)	90(30.20%)	χ² = 1.035	0.021

**Table 3:** Comparison of postoperative results between the two groups

	Hemiarthroplasty group (n=306)	Constrained acetabular liners group (n=298)	t-value, χ² value	P
HHS scores				
12 months follow up	82.10±3.50	79.55±4.15	t = 2.603	＜0.001
24 months follow up	84.25±3.35	83.75±2.85	t = 1.215	0.852
Last follow up	85.80±2.65	85.05±3.25	t = 0.972	0.613
VAS score				
Preoperation	6.32±0.56	6.05±0.26	t = 0.807	0.713
12 months follow up	1.85±0.55	1.59±0.38	t = 0.235	0.105
24 months follow up	1.02±0.75	1.15±0.25	t = 0.502	0.681
Last follow up	1.05±0.25	1.08±0.32	t = 0.386	0.192
WOMAC scores				
12 months follow up	33.56±6.76	35.27±3.86	t = 2.732	0.915
24 months follow up	30.08±3.55	31.95±4.28	t = 1.105	0.602
Last follow up	28.75±5.15	30.15±7.25	t = 0.535	0.296
Forgotten Joint Score (FJS)				
12 months follow up	71.96±6.04	70.85±7.09	t = 0.212	0.353
24 months follow up	75.80±5.59	73.86±6.85	t = 0.365	0.127
Last follow up	82.24±6.77	80.62±3.15	t = 0.215	0.528

Postoperative clinical scores, including HHS, VAS, and FJS, showed no significant differences between groups. However, a significant difference existed in HHS scores at 12 months postoperatively (82.10 ± 3.50 vs. 79.55 ± 4.15, *P* < 0.001) (Table 4).

**Table 4:** Radiological indices of prosthesis position in hemiarthroplasty group and constrained acetabular liners group

	Hemiarthroplasty group (n=306)	Constrained acetabular liners group (n=298)	t-value, χ² value	P
Leg length discrepancy (mm)	5.02±2.07	5.15±3.32	t = 0.831	0.131
Offset (mm)	44.59±6.97	43.55±5.02	t = 3.252	0.341
Femoral stem anteversion	14.25±10.20	15.55±11.80	t = 1.069	0.720
Femoral stem coronal alignment				
Valgus	56(18.30%)	63(21.14%)	χ² = 0.952	0.080
Neutral	230(75.16%)	216(72.48%)		
Varus	20(6.54%)	19(6.38%)		

### Radiological measurements

No statistically significant differences were observed between groups in radiological characteristics, including LLD, femoral offset, femoral stem anteversion, and femoral stem coronal alignment. Patients’ radiological measurements are summarized in Table 5.

**Table 5:** Multivariable analysis of complications

Complications	Odds ratio	95% CI	P
Dislocation	0.120	0.022-0.552	0.007
Aseptic loosening	1.155	0.708-1.782	0.045
Heterotopic ossification	1.198	1.020-1.850	0.043

*CI* confidential interval

## Discussion

This study is among the few comparative analyses evaluating constrained liner total HA versus conventional hemiarthroplasty in elderly patients with neurological disorders and unstable comminuted femoral ITF. Neurological diseases predispose patients to ITF due to reduced muscle strength and balance impairment; however, the optimal surgical approach for these fractures in elderly populations remains unclear [16]. Most researchers advocate hemiarthroplasty for elderly patients with unstable ITF, involving replacement of the femoral head with a prosthetic implant [17]. Proponents of total HA (THA) suggest it may provide superior patient function and quality of life compared to hemiarthroplasty [18]. However, THA raises concerns related to potentially increased surgical morbidity compared with hemiarthroplasty [19]. THA is associated with higher blood transfusion rates, longer operation times, extended hospital stays, and increased risks of perioperative complications, infection, readmission, and mortality [20–22]. Moreover, elderly patients with neurological disorders undergoing THA face an increased risk of dislocation, often necessitating revision or additional interventions [23, 24]. Appropriate implant selection is essential to enhance prosthetic stability and minimize risks of instability or failure postoperatively [25]. In orthopedic patients with compromised muscle function due to severe muscle atrophy, paralysis, or neuromuscular dysfunction, constrained acetabular devices have successfully improved prosthetic stability and reduced postoperative dislocation [26, 27].

Hemiarthroplasty is a simpler surgical procedure focusing exclusively on the femoral side of the hip joint [28]. In contrast, THA requires additional acetabular interventions, which extend surgical duration and increase intraoperative blood loss [29]. Significant differences in Harris Hip Scores (HHS) were noted between groups during the early postoperative period, particularly within the first year. However, no differences in long-term HHS scores were observed between the two groups. Extensive patient participation in rehabilitation exercises contributed significantly to successful hip functional recovery, as indicated by this finding [30].

Hip dislocation after THA is a frequent postoperative complication, often requiring revision surgery [31]. Neuromuscular disorders and muscle weakness are acknowledged as risk factors increasing postoperative dislocation [32]. Maintaining optimal hip positioning after surgery is challenging in these patients, potentially leading to early postoperative dislocation [33]. Careful preparation is necessary to prevent dislocation when performing THA in these patients. Some researchers suggest that the acetabular cup used in hemiarthroplasty enhances joint stability and range of motion due to a polyethylene bearing, offering a larger femoral head and increased jump distance from an improved head-neck ratio [34, 35]. Considerable evidence indicates THA causes more trauma to external rotator muscles and posterior joint capsule compared to traditional hemiarthroplasty, significantly increasing dislocation risk [36]. However, posterior capsule and external rotator muscle repair, combined with increased acetabular cup anteversion, can significantly reduce dislocation rates with posterior approaches [37, 38]. In our study, dislocation rates were 1.01% in the CAL group and 8.50% in the hemiarthroplasty group. Previous studies have reported dislocation rates of 5% to 8% annually in patients with neuromuscular disorders such as cerebral palsy, muscular dystrophy, dementia, and Parkinson’s disease [39–41], consistent with our findings. Thus, constrained liner THA appears effective in lowering dislocation risk.

In our study, HO was significantly more common in the CAL group than in the hemiarthroplasty group. Prior studies indicate a close relationship between HO formation and subfascial hematoma [42]. Hematoma formation triggers the release of osteogenic factors, subsequently influencing HO development [43, 44]. Compared with hemiarthroplasty, THA involves acetabular preparation, causing greater soft tissue trauma, which may increase hematoma formation and HO risk [45]. Accumulating evidence demonstrates soft tissue injury can stimulate fibroblast proliferation and extracellular matrix accumulation [46, 47], eventually resulting in HO via endochondral ossification [48]. Individuals with a history of cerebrovascular accidents face elevated HO risk after THA [49, 50]. DiCaprio et al. [51] reviewed 31 THAs in 22 stroke patients, identifying HO in 36% compared to 15% in a control group. Thus, prophylactic strategies, including pharmacological interventions or physical therapy, should be considered in THA patients with cerebrovascular histories to minimize HO risk and optimize postoperative outcomes. None of the patients in our study received routine HO prophylaxis, such as non-steroidal anti-inflammatory drugs or local radiotherapy, thus eliminating potential confounding factors and ensuring reliable statistical outcomes.

Extensive research indicates patients with neurological disorders affecting the hip encounter specific challenges in HA. Muscle weakness, rigidity, tightness, and tremors cause uneven hip muscle tone, potentially leading to hip dislocation and aseptic loosening [52, 53]. In our study, the constrained acetabular liner (CAL) group exhibited a higher rate of aseptic loosening compared to the hemiarthroplasty group. While constrained acetabular cups enhance hip stability, they restrict joint motion [54]. This restriction results in component impingement, excessive wear-particle generation, and macroscopic implant damage [55]. Increased mechanical stress on the liner further accelerates early implant failure and loosening [56, 57]. All enrolled patients completed a 6-year follow-up, enabling a reliable evaluation of long-term prosthetic complications. These patients had multiple comorbidities associated with an elevated risk of implant loosening [58]. Contributing factors include poor bone quality and reduced bone mineral density (BMD) [59, 60]. Osteoporosis impairs initial implant fixation, induces micromotion, inhibits osseointegration, and triggers chronic inflammation [61–63]. Additionally, nutritional deficiencies, iron overload, and hyperglycemia adversely affect bone-implant integration [64, 65]. Preoperative risk assessment and long-term monitoring are crucial to prevent implant loosening and manage bone resorption progression. Conditions such as diabetes, osteoporosis, and chronic inflammatory disorders negatively affect bone remodeling and compromise implant fixation. It is vital to optimize metabolic control, manage BMD, and coordinate perioperative care, including consultations with endocrinology and rheumatology specialists, and address urinary infections to maintain bone quality and reduce related risks.

CAL can reduce dislocation by containing the femoral head beyond its equator, thereby preventing dislocation. However, constrained liners substantially restrict the primary arc range, potentially increasing instability risk paradoxically. Poor initial positioning can lead to liner dissociation, component loosening, and recurrent dislocations, resulting in a high failure rate [66]. The management of aseptic loosening depends on the extent of periprosthetic bone loss around femoral and acetabular components. Femoral reconstruction implant choice depends on residual metaphyseal and diaphyseal bone stock and includes proximally coated stems, fully coated stems, and fluted tapered stems. In cases of severe bone loss, a megaprosthesis or impaction bone grafting is recommended. Standard cups are sufficient for mild acetabular bone deficiency. For inadequate structural support at the acetabular rim, acetabular augments, jumbo cups, or cup-cage constructs are recommended alternatives [13].

Several limitations of this study must be acknowledged. First, the data were observational and retrospective, inherently increasing the risk of selection bias. All included studies were assessed for bias risk. Additionally, this research was conducted at a single medical center, limiting the generalizability of findings to other populations or clinical settings. Second, the same surgical team performed all procedures, making it impossible to evaluate individual surgeon proficiency’s influence on outcomes. Third, multiple factors contribute to dislocation, aseptic loosening, and HO after HA. Implant selection alone is unlikely the sole determinant of dislocation or revision necessity. Additionally, continuous improvements in surgical techniques and implant designs may influence the interpretation and generalizability of these results.

## Conclusion

Current findings suggest constrained liner total HA in patients with neurological disorders undergoing HA for unilateral unstable comminuted femoral ITF is associated with a lower postoperative dislocation rate but increases the risk of aseptic loosening and HO. Although constrained liners reduce dislocation risk, their overall clinical benefit appears limited in this patient population. Additionally, no significant differences in long-term hip function outcomes were observed between constrained liner total HA and conventional hemiarthroplasty.

## Supplementary Information

Supplementary Material 1.

## Data Availability

All the data generated or analyzed during this study are included in this published article.

## Abbreviations

THA: Total hip arthroplasty
CAL: Constrained acetabular liners
ITF: Intertrochanteric fractures
HO: Heterotopic ossification
IF: Internal fixation
HA: Hip arthroplasty
HHS: Harris Hip Joint Score
VAS: Visual Analogue Scale
FJS: Forgotten Joint Score
Hb: Hemoglobin
CT: Computed tomography
LLD: Leg length discrepancy
IRB: Institutional Review Board
