# Supplementary material for: Constrained acetabular liner total hip arthroplasty versus conventional hemiarthroplasty for unstable comminuted femoral intertrochanteric fractures in elderly patients with neurological disorders
Source: BMC Surg. 2026 Jun 23;26:609. doi: 10.1186/s12893-026-03973-2 (PMC13563775; doi:10.1186/s12893-026-03973-2)
Supplement: Supplementary file 1 [file 12893_2026_3973_MOESM1_ESM.pdf]

YIMORE

EDITING CERTIFICATE

To whom it may concern:

This document is to confirm that *Zibo Yimore Translation CO. LTD* happily provided English proofreading services for the following thesis.

Topic: Constrained Acetabular Liner Total Hip Arthroplasty versus Conventional Hemiarthroplasty for Unstable Comminuted Femoral Intertrochanteric Fractures in Elderly Patients with Neurological Disorders

Author: Mingming Dong, M.D, Yandan Liu, Congcong Wei, M.D.

We performed comprehensive language editing, including corrections in grammar, punctuation, and syntax to the best of our abilities. The revised document was returned to the author on 05/28/2026. We are not aware of any changes made to the manuscript after this date and have kept a copy of the original file for any potential legal dispute.

Sincerely,

Henry Graff

Zibo Yimore Translation CO. LTD

Tel: 0533-2775538 Email: yimorezhls@163.com
